# The Categorization of Perinatal Derivatives for Orthopedic Applications

**DOI:** 10.3390/biomedicines12071544

**Published:** 2024-07-11

**Authors:** Amol H. Trivedi, Vicki Z. Wang, Edward J. McClain, Praveer S. Vyas, Isaac R. Swink, Edward D. Snell, Boyle C. Cheng, Patrick J. DeMeo

**Affiliations:** 1 Orthopaedic Institute, Allegheny General Hospital, Allegheny Health Network, Pittsburgh, PA 15212, USA; aht56@drexel.edu (A.H.T.); vicki.wang@ahn.org (V.Z.W.); edward.mcclain@ahn.org (E.J.M.IV); praveer.vyas@ahn.org (P.S.V.); isaac.swink@ahn.org (I.R.S.); edward.snell@ahn.org (E.D.S.); patrick.demeo@ahn.org (P.J.D.); 2 Drexel University College of Medicine, Drexel University, University City Campus, Philadelphia, PA 19104, USA

**Keywords:** placenta, perinatal derivative, biologics, orthopedics, musculoskeletal pathology

## Abstract

Musculoskeletal (MSK) pathology encompasses an array of conditions that can cause anything from mild discomfort to permanent injury. Their prevalence and impact on disability have sparked interest in more effective treatments, particularly within orthopedics. As a result, the human placenta has come into focus within regenerative medicine as a perinatal derivative (PnD). These biologics are sourced from components of the placenta, each possessing a unique composition of collagens, proteins, and factors believed to aid in healing and regeneration. This review aims to explore the current literature on PnD biologics and their potential benefits for treating various MSK pathologies. We delve into different types of PnDs and their healing effects on muscles, tendons, bones, cartilage, ligaments, and nerves. Our discussions highlight the crucial role of immune modulation in the healing process for each condition. PnDs have been observed to influence the balance between anti- and pro-inflammatory factors and, in some cases, act as biologic scaffolds for tissue growth. Additionally, we assess the range of PnDs available, while also addressing gaps in our understanding, particularly regarding biologic processing methods. Although certain PnD biologics have varying levels of support in orthopedic literature, further clinical investigations are necessary to fully evaluate their impact on human patients.

## 1. Introduction

Musculoskeletal (MSK) pathology includes a wide spectrum from mild discomfort to irreversible injuries involving muscles, tendons, bones, cartilage, ligaments, and nerves [1]. These conditions affect approximately 127 million people in the United States, representing roughly 1 in every 3 individuals [2]. In 2019, they ranked as the third leading cause of disability-adjusted life years, contributing to 25.7% of the total years of life lost to disability [2]. The profound impact of these pathologies has ignited growing interest in the search for more effective treatments, particularly within the field of orthopedics.

**Figure 1:**
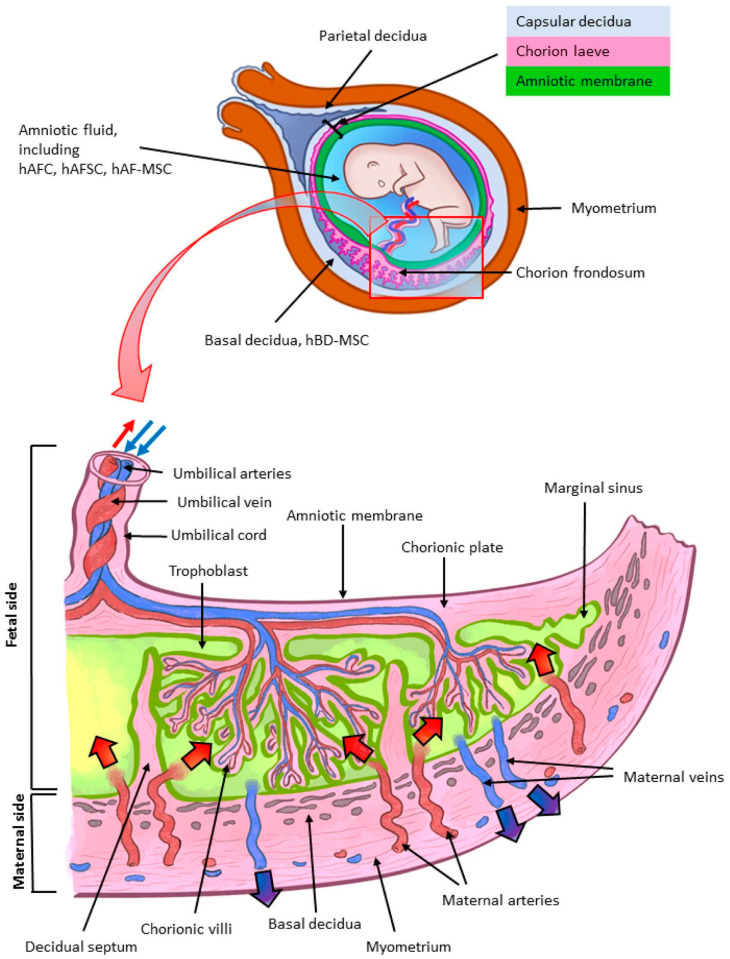
Anatomic depiction of a full-term human placenta and the fetus. The interactions between the maternal side and fetal side of the placenta are specifically illustrated in the magnified portion. This figure is credited to [3]; https://creativecommons.org/licenses/by/4.0/ accessed 30 May 2024; no alterations were made to the original figure.

The human placenta has emerged as a focal point for biologics within the realm of regenerative medicine. Serving as a vital organ of gestation, it facilitates the delivery of oxygenated blood and nutrients from the maternal supply to the developing fetus while filtering out harmful waste products [3]. The anatomy of the placenta, characterized as a disc-shaped organ with a multi-layered placental membrane encasing the fetus, offers valuable insights into its function [4]. (Figure 1) Among its components, the human amniotic membrane (hAM) is the innermost layer, characterized as a multilayered structure rich in collagen, fibronectin, and laminin [5]. Additionally, its stromal layer contains interstitial collagens (types I and III), providing mechanical strength, alongside type IV and V collagen, which establish filamentous connections [5]. Notably, it contains fetal hyaluronic acid (HA), which suppresses the expression of inflammatory factors (TGF-B1, -B2, and -B3) and TGF receptors, exerting an anti-fibrinogenic effect [6].

Surrounding the umbilical cord—comprising one vein and two arteries—is the avascular amniotic membrane, within which resides Wharton’s jelly [5]. Composed of HA and chondroitin sulfate (CS), Wharton’s jelly provides structural support to prevent compression or torsion of the umbilical vessels [7,8]. Notably, it harbors the highest concentration of mesenchymal stem cells among bodily tissues, along with fibroblast-like and myofibroblast-like cells [5,7]. Furthermore, growth factors (GFs) such as insulin-like growth factor binding proteins (IGFBPs) and transforming growth factor alpha (TGF-a) have been identified [7]. Additionally, pro-inflammatory cytokines like macrophage colony-stimulating factor (MCSF) and anti-inflammatory cytokines like interleukin 1 receptor antagonist (IL-1RA) are present [7].

Subsequently, an intermediate spongy layer separates the amnion from the chorion, featuring a meshwork of collagen, proteoglycans, and glycoproteins supporting amniotic movement [5]. Adjacent to this layer lies the chorion on the maternal side, comprising a reticular layer, a basement membrane, and a trophoblast layer communicating with the uterine decidua [5]. While it is up to four times thicker than the amniotic membrane, the chorion lacks nidogen and matrix metalloproteinases (MMP)-1 and MMP-2 compared to the amniotic membrane, albeit it shares similar extracellular matrix (ECM) components such as collagen (types I, III, IV, V, and VI), fibronectin, and laminin [9,10]. Furthermore, the amniotic membrane forms the sac housing the fetus, which is bathed in human amniotic fluid (hAF) [11]. Predominantly composed of electrolytes and water, hAF also contains HA and antimicrobial substances such as alpha-defensins, lactoferrin, and lysozyme [5,12]. Moreover, TGF-a, TGF-B1, insulin-like growth factor I (IGF-I), and high levels of epidermal growth factor (EGF) have been identified in hAF [6].

The use of placental tissues for clinical applications dates back to the early 20th century [4]. With their increasing utilization today, biologics derived from the placenta, referred to as perinatal derivatives (PnDs), can be sourced from its components, each possessing unique properties. Based on the previous description of perinatal tissue, they can be thought of as encompassing three features: the extracellular matrix, a variety of growth factors, and regenerative cellular activity (likely through growth factors that bring about this activity). It is the combination of a scaffold and growth factors that makes PnDs suitable for clinical application. The extracellular matrix of PnDs serves as a scaffold for endogenous cell growth, in addition to conferring mechanical strength and elasticity to the product or graft. Growth factors present in the matrix may facilitate tissue development by stimulating the recruitment, differentiation, and proliferation of endogenous cells. The proteins contained in PnDs are an essential part of communication with the host tissue, which includes modulation of the appropriate healing response through cell signaling [13]. However, the variation in tissue processing can impact the structural and biological characteristics of PnDs [5].

PnD biologics with viable cells represent a form of this variation. Placental cells can modulate immune responses through trophic factors, stimulating parenchymal cells to initiate repair in a paracrine manner [5,14]. While cellular biologics exist, there is also a different group of acellular, decellularized, and/or non-viable PnD biologics that undergo unique processing. Decellularization involves the removal of native cells from living tissues biologically, chemically, and/or physically, leaving a three-dimensional ECM scaffold [15]. This process eliminates immunogenic components, thereby averting potential immune responses in clinical applications [15]. Achieving a balance between cellular removal and ECM preservation is crucial to optimizing healing outcomes [5].

With a burgeoning interest in research on PnDs, the International Network for Translating Research on Perinatal Derivatives into Therapeutic Approaches (SPRINT) was established to streamline research efforts in the field. Funded by the European Cooperation in Science and Technology (COST), a consensus paper by Silini et al. delineated human placental tissue and cell nomenclature, providing a guiding framework for the terminology used in this review [3,16]. There have been a series of review articles as part of this initiative which discuss the potential of perinatal stem cells in medicine [3]. However, much of this research is still in the translational stage of development. The overarching aim of this review is to elucidate our understanding of the PnD allografts (i.e., amniotic membranes, chorionic membranes, etc.) that are currently available for clinical use and present current evidence that may benefit orthopedic surgeons in determining how these products may help in treating various MSK pathologies.

## 2. Wound

Successful wound healing is of prominent importance for orthopedic surgery, as subpar healing of the surgical site can lead to detrimental complications [17]. Some surgical wounds may even progress to chronic wounds that persist without healing for weeks, months, or even years [18]. Therefore, there is a clear need in orthopedics for therapies that actively accelerate the healing of the incisional wounds left at surgical sites.

Much of the following background on wound healing is a brief summary from Cañedo-Dorantes [19]. Successful wound healing occurs in three main phases: inflammation, proliferation, and remodeling. Inflammation starts within seconds of skin injury and peaks during the proliferation phase. In proliferation, granulation tissue takes over the wound site, with prominent amounts of fibroblasts, granulocytes, macrophages, and blood vessels [20]. The final phase involves remodeling the tissue created during the proliferation phase and can last for months following the injury. The following sections will go more in depth into the mechanisms and pathways involved in skin healing.

After the initial acute inflammatory response, platelets release TGF-β, which recruits various important immune response cells, such as neutrophils and macrophages, and their cell–cell interactions are involved in the release of important growth factors in the wound healing process, including platelet-derived growth factor (PDGF), TGF-β1, and vascular endothelial growth factor (VEGF). Tissue-resident macrophages direct monocytes to the wound site and release chemoattractants that recruit more neutrophils, which in turn recruit inflammatory monocytes that mature into macrophages [20]. Tumor necrosis factor-alpha (TNF-α) induces macrophages toward the M1 phenotype, which promotes inflammation. TNF-α also activates the prototypical pro-inflammatory signaling pathway, nuclear factor-κB (NF-κB). Excessive activation of NF-κB is implicated in inflammatory diseases and metabolic disorders [21].

Fibroblast proliferation is one of the identifying characteristics of the proliferation phase as they respond to cytokines and growth factors to promote collagen development and wound contraction. Keratinocytes and the re-epithelialization process are additional markers of this phase. Assuming proper healing, the wound environment changes from pro- to anti-inflammatory. Macrophages turn into the M2 phenotype, which has an anti-inflammatory effect through the release of the IL-10 cytokine and inhibition of the production of IL-1β and TNF-α. Lymphocytes promote the production of interferon-gamma (IFN-γ), which causes macrophages and fibroblasts to secrete greater levels of VEGF and TGF-β. These molecules encourage wound healing through collagen deposition, angiogenesis, and suppressing a further inflammatory response. Endothelial cells undergo a metabolic switch in the hypoxic conditions of a wound. This switch, along with proangiogenic factors like VEGF, fibroblast growth factor 2 (FGF2), PDGF, and TGF-β1, initiates neovascularization. Increased VEGF expression, in addition to macrophage presence, guides the differentiation of endothelial cells into these metabolic subtypes. In the final remodeling phase, granulation tissue is replaced by mature tissue. MMPs and macrophages replace type III collagen with type I collagen, which continues to be reorganized until the wound heals into a low-cellularity scar. Post-surgical complications at the skin level include infection of the surgical site and potential progression into a chronic wound [22]. Bacteria induce an overly activated response of the inflammatory system, using neutrophils and factors such as TNF-a, inhibiting proper healing through breakdown of the ECM to prevent proper remodeling into mature tissue [23,24,25].

PnDs have been studied and shown to positively impact many factors in the wound healing process. They are an excellent allograft material, as their low levels of human leukocyte antigens ensure the tissue’s low immunogenicity [26]. The most common form of PnDs used in wound healing applications is the amniotic membrane, isolated from the other tissues, cleaned, and used fresh/cryopreserved or after decellularization techniques [15]. However, some studies have also gleaned the impact of human chorion membranes (hCMs) on healing, as well as PnDs distributed in the form of hydrogels or lyophilized and seeded in scaffolds. In the context of wound healing, PnDs are antibacterial and anti-inflammatory, function as an excellent ECM scaffold, promote angiogenesis, and induce rapid re-epithelialization.

Although the cells contained in the amniotic membrane and related perinatal derivatives confer significant antibacterial activity in allografts, decellularized hAM (dhAM) still holds significant antibacterial properties pertinent to surgical wounds. The ECM components hyaluronic acid, fibronectin, collagen, laminin, and vitronectin have shown antimicrobial action against *S. aureus*, *E. coli*, *P. aeruginosa*, *Streptococcus*, *E. faecalis*, *S. mutans*, *P. gingivalis*, *P. oris*, *A. actinomycetemcomitans*, and *P. acnes* [27].

PnDs’ ameliorative effect on wound site inflammation has been well documented. Decellularized human amnion/chorion membrane (dhACM) contains anti-inflammatory cytokines IL-4 and IL-10. It also contains IL-6, which can act as a pro- or anti-inflammatory agent depending on the context [28]. IL-4 polarizes Th cells into Th2 effector cells and propagates Th2 responses. IL-10 works to subdue inflammation mostly through its suppression of IL-1, IL-6, IL-12, and TNF, along with other pro-inflammatory chemokines [29]. Besides its role as an antibacterial agent, secretory leukocyte protease inhibitor (SLPI) also protects local tissue against the detrimental effects of inflammation through protease inhibition, namely neutrophil elastase inhibition. SLPI also defends against inflammation by downregulating the macrophage response via inhibition of the NF-κB pathway [30]. If implanted under skin flaps, the amniotic membrane provides a buffer that prevents leukocytes from infiltrating the skin flap, dampening the injury cascade that would otherwise induce inflammation and necrosis.

The hAM and hCM are structurally similar to the ECM and provide a good scaffold for fibroblasts to attach to and proliferate on. dhAM has been characterized as 90% composed of ECM molecules—for example, fibronectin, a key molecule for fibroblast adhesion [31]. dhACM also contains tissue inhibitors of metalloproteinase 1 (TIMP1), 2, and 4, which help to mitigate the ECM-degrading functions of MMPs [28]. It is important to note that fibroblast proliferation may vary based on whether the cells are exposed to the epithelial or stromal side of the amniotic membrane, with the stromal side resulting in more favorable growth characteristics [32].

It is worthy of mentioning that the impact of the hAM on angiogenesis is context-dependent—hAM acts as an agent of anti-angiogenesis in ophthalmological applications but of pro-angiogenesis in cutaneous lesions [33,34]. However, in the context of post-surgical incisional wounds, the pro-angiogenic effect of perinatal derivative therapies is helpful to the healing process. Angiogenic growth factors can be found in dhACM samples, and their mechanisms are elucidated in Table 1 [28]. dhACM also contains the chemokine IL-8, whose many functions include angiogenesis [35]. IL-8 is thought to enhance the proliferation and survival of CXCR1- and CXCR2-expressing endothelial cells, but its mechanism in angiogenesis is not well understood [36].

PnDs have been shown to contribute significantly to re-epithelialization through impacting the function of keratinocytes and inducing various pathways in wound contraction. The amniotic membrane secretes cytokines that modulate TGF-β signaling in the keratinocytes and stimulates c-Jun expression via phosphorylation of the SAP and JNK kinases [46]. c-Jun’s impact on wound closure was confirmed in mice models that showed impaired keratinocyte migration under c-Jun knockout conditions [47]. The presence of dhACM also induced greater expression of paxillin, a focal adhesion adapter protein, thereby inducing cytoskeletal changes in focal structures that promote cell migration and wound restoration [48,49]. dhAM also induces greater a-SMA secretion by the myofibroblasts, aiding in wound contraction [50]. Mesenchymal progenitor cell recruitment is also increased in the presence of dhACM—MSCs play a role through all stages of wound healing, acting to downregulate inflammation through TNF-α suppression, as well as IL-4 and IL-10 production in the acute phase; produce growth factors like VEGF, HGF, and PDGF while recruiting cells like keratinocytes and fibroblasts during proliferation; and finally regulate MMPs/TIMPs and collagen deposition during remodeling [35,51].

Many in vitro, in vivo, and clinical studies have strongly supported the ability of perinatal derivatives to hasten successful wound healing better than standard-of-care practices. 

### 2.1. Preclinical Evidence

Experiments evaluating fibroblasts seeded and cultured on PnD products proved the effects of these membranes on angiogenesis, inflammation, and epithelialization. Fibroblasts showed greater proliferation in a dhACM environment and produced more MCP-1, IL-8, and fibronectin [31,35]. Other cell type experiments also showed positive evidence. In experiments with tenocytes, which are tendon fibroblast cells, the pro-inflammatory cytokine TNF-α was downregulated [52]. Monocyte adhesion was reduced in AM-seeded HUVECs in a TNF-α-stimulated inflammatory environment, and VCAM-1 and ICAM-1 genes were downregulated [33].

In vivo experimentation has continued to prove the beneficial effects of PnDs on wound healing. dhACM treatment promoted angiogenesis across various rat studies, increasing the vascular branch number and length in skinfold chamber models and dorsal incision/skin flap experiments [24,53]. Other rat studies also proved PnDs’ anti-inflammatory effect on wound healing. Surgically induced full-thickness skin defects treated with dhAM showed a lower white blood cell count and lower expression of TGF-β1, indicating a reduced inflammatory response [50]. An amnion hydrogel implanted subcutaneously resulted in a lower CCR7 density, blocking immune cell recruitment [54,55]. Work has also been undertaken to glean the specific effects of individual beneficial components of the amniotic membrane. Treatment with SLPI in a rat dorsal incision model showed a reduction in TNF-α expression and an increase in the quantity of new blood vessels formed at the wound site [56].

### 2.2. Clinical Evidence

A number of clinical studies have elucidated the positive effect of PnD therapies on wound healing. Ilic et al. conglomerate and summarize several separate studies involving decellularized amnion treatment for diabetic foot ulcers, with compelling evidence showing that application of a perinatal derivative led to greater success in reducing the wound size and closing chronic wounds than standard-of-care practices [57]. Deep-trauma-induced wounds with patent retard in epithelialization saw vast improvements and even total wound closure after the application of amniotic membrane [47]. The largest single-center study to date treated chronic wounds of various etiologies with dhACM and demonstrated significant healing {≥70% reduction in the wound surface area} in three-quarters of wound incidences [58]. Fairbairn also reviewed the results of studies using various forms of human amnion for various etiologies of skin wounds, showing that amnion induces faster epithelialization in burns and chronic wounds, and in vaginal reconstruction, complete epithelialization was completed within 8 weeks in the presence of amniotic membrane [59]. In comparison with cryopreserved allografts in the treatment of genital burns, dhACM therapy again resulted in faster epithelialization [60]. dhACM proved to aid in wound healing even in elderly patients—weekly application of dhACM grafts onto full-thickness surgical defects in elderly patients post Mohs surgery induced complete epithelialization without the need for bone chiseling to stimulate pinpoint bleeding and granulation over the exposed bone [61]. Besides hAM, hCM was also used to treat cases of gingival recession, with significant results in root coverage and thickening of the gingival biotype [62]. 

### 2.3. Summary of PnDs in Wounds

In the literature, most of the discussion surrounding wound healing and PnDs focuses on subcutaneous grafting. However, they could be particularly useful if applied as membranes over exposed subcutaneous tissue in instances where skin coverage is lost, such as due to trauma or wound dehiscence. Further, most clinical studies have applied hAM to non-healing wounds, but its universal benefit in healing processes suggest that using hAM for the treatment of the incisional wounds incurred by orthopedic surgery would likely help to fend off infection, reduce inflammation, and speed up tissue regeneration. Therefore, it would be beneficial to see studies investigating PnDs and their impact on incisional and traumatic wounds, as their underlying healing potential has shown promise.

## 3. Tendinopathy

Tendon injuries and disorders are common and lead to significant disability and pain, with over 30 million tendon-related procedures taking place annually worldwide [63,64]. Specifically, we will focus on the commonly injured flexor tendon, the Achilles tendon, and the rotator cuff tendons. Thomopoulos et al. described tendon injury in detail, and their discussion has been summarized here. First, flexor and rotator cuff tendons are both intra-synovial and lack spontaneous healing, while the Achilles tendon is extra-synovial in nature and involves fibrous tissue formation after injury. Therefore, the ability of a tendon to heal is impacted by the location of that tendon, as well as its biological environment [63]. 

Normally, tendon healing follows a typical course of early inflammation, followed by proliferation and ending with remodeling. The essence of tendinopathy is a failed healing response, with degeneration and haphazard proliferation of tenocytes, disruption of collagen fibers, and a subsequent increase in the non-collagenous matrix [65].

Recent evidence suggests that early modulation of the inflammatory process after tendon repair, fibroblasts, and tenocytes may lead to improved healing [63,66,67]. This includes influencing both pro-inflammatory and anti-inflammatory factors. For instance, M1 macrophages are pro-inflammatory and are well understood to be stimulated by bacterial signals or type 1 helper cell (Th1) cytokines. Subsequently, by releasing interleukin-1β (IL1β), interleukin-12 (IL12), tissue necrosis factor-α (TNFα), and others, these biomolecules create a pro-inflammatory state which can lead to scarring and fibrosis. On the other hand, M2 macrophages are induced by type 2 helper cell (Th2) cytokines. This creates an anti-inflammatory response through the release of interleukin-10 (IL10) and transforming growth factor-β1 (TGFβ1), which effectively clear excess ECM in scarring [63]. During the remodeling phase, several growth factors and regulators, such as platelet-derived growth factor (PDGF), basic fibroblast growth factor (bFGF), TGF-β, and vascular endothelial growth factor (VEGF), play crucial roles. Further, the natural expression patterns of these factors vary dramatically over time during tendon healing. As a result, the manipulation of these growth factors during this process is the key to enhancing tendon repair [63]. 

### 3.1. Preclinical Evidence

The current literature on perinatal derivatives (PnDs) has given us a glimpse into their ability to influence key contributors and phases of tendon healing. Specifically, there have been in vitro studies that lend support to their inherent healing potential. McQuilling et al. conducted an in vitro study to understand the tenocyte response to hypothermically stored hAM (HS-hAM) and dehydrated hACM. Both these PnDs released familiar growth factors that are important to tendon healing such as PDGF, b-FGF, and insulin-like growth factor I (ILGF-1). Further, they both promoted tenocyte proliferation and migration and modulated the inflammatory response. Treating tendons with HS-hAM resulted in decreased production of pro-inflammatory molecules such as matrix metalloproteinase-1 (MMP-1) [68].

Other forms of PnDs are connective tissue matrices (CTMs). These are distinct “scaffold particulates” that offer unique structural, biochemical, and immunogenic properties [66]. In vitro studies conducted by Mao et al. showed that CTMs have the potential to mitigate de-differentiation, inflammation, and adhesion formation and expedite tendon healing. Their study compared various types of CTMs (minimally manipulated, decellularized flowable, and liquid) and their impact on the different biomolecular aspects of tendon healing. The investigators found that the decellularized flowable matrix promoted the highest number of viable tenocytes, showed significant tenocyte growth, and released factors which promoted tenocyte migration. Under inflammatory conditions, the expression of pro-inflammatory molecules was lower when compared to the minimally manipulated matrix and promoted the expression of anti-fibrotic growth factors such as TGF-*β*3 [66].

**Figure 2:**
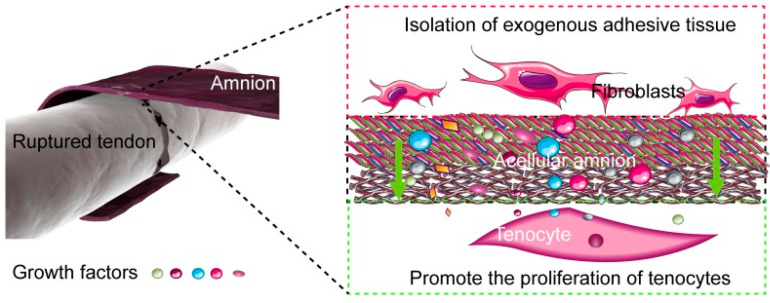
Amniotic membrane wrap for tendon rupture. Magnified image describes the membrane’s endogenous healing properties with factor recruitment and tenocyte proliferation. Additionally, the wrap serves as a protective physical barrier. This figure is credited to [69]. https://creativecommons.org/licenses/by/4.0/ accessed 30 May 2024; no alterations were made to the original figure.

Additionally, there have been studies in animal models that have built upon what we have come to understand at a basic science level. PnDs in tendon healing, specifically within the flexor tendon, the Achilles tendon, and the rotator cuff tendons, have been investigated through in vivo studies. For instance, Sang et al. assessed the healing in the flexor digitorum profundus (FDP) by inducing tendon injury models in 30 chickens on the ipsilateral third toe. Those in the experimental group had an acellular hAM wrapped around the location of the stitched tendon, while the control group received no treatment. Tendons with hAM showed an increased number of tenocyte nuclei and a larger area of collagen I and fibronectin on fluorescence staining. On adhesion evaluation at 2, 4 and 6 weeks, tendons with hAM healed well without adhesions and were more like their native structure, which contrasted with the fibrotic tendons that were found in the control group. Lastly, biomechanical testing at weeks 2 and 4 showed the hAM tendons to have a significantly higher maximal tensile strength, although the investigators reported an insignificant difference in strength at week 6 between the groups [69] (Figure 2).

Similarly, Liu et al. studied the healing of flexor tendons and tendon sheaths by creating injury in these structures within the third toe of 60 chicken models. Investigators evaluated the outcomes of the chickens within three groups (dhAM group, absorbable medical membrane (MM) group, and control group) over the course of the study. On electron microscopy, the tendon cells proliferated, and the hAM group had a higher number of synovial cells. Histological evaluation at 8 and 12 weeks revealed that the fibrocytes in the hAM and MM groups were arranged in a dense and layered manner when compared to the disordered distribution of fibrocytes in the control group. Finally, biomechanical evaluation showed a higher tendon sliding distance, total flexion angle, and maximum tensile strength in the hAM and MM groups when compared to the control [70].

McQuilling et al. conducted a study assessing how dehydrated hACM can impact tendon repair in a diabetic model to reflect the increasing incidence of this impaired healing population. A full-thickness injury was created in the Achilles tendon in 30 rat models with type 2 diabetes mellitus (T2DM). These tendons were either wrapped with dehydrated hACM or not wrapped at all (control group). On both day 14 and day 28, the hACM tendons yielded an increased cell density and superior tendon fiber organization when compared to the unwrapped tendons. Further, the hACM tendons had a significantly higher number of cells upon quantification when compared to the unwrapped tendons at days 14 and 28. The biomechanical testing results yielded similar trends, with the hACM tendons showing significant increases in maximum tensile load and stiffness when compared to the controls. To study the impact of diabetes through impaired healing, investigators included a non-injured sham group. When compared to the sham group, the injured groups were both structurally inferior and had a substantial deficit in mechanical strength [68].

Finally, a rabbit model study by Yuan et al. focused on the rotator cuff tendons. These tendons, known for their limited self-healing ability, have attracted attention for potential biologic augmentation. In this study, researchers induced supraspinatus tendon injuries in 54 rabbits, dividing them into either a scaffold repair group or a non-scaffold control group. The scaffold, derived from decellularized human umbilical cord Wharton’s jelly (dhUC-WJ), retained glycosaminoglycans (GAGs) and collagens crucial for maintaining structural integrity. Ultimately, the dhUC-WJ group exhibited significantly enhanced proliferation, migration, viability, and growth of rabbit tendon stem/progenitor cells compared to the control group. Moreover, the dhUC-WJ scaffold facilitated the formation of continuous and organized tendon repair compared to the disordered tendons observed in the control group. Biomechanical analysis revealed that the dhUC-WJ group consistently demonstrated significantly higher maximum failure loads at weeks 4, 8, and 12 post-surgery compared to the control group. Additionally, the dhUC-WJ-treated tendons closely resembled the native supraspinatus tendon and exhibited a significantly higher tensile modulus compared to the control group [71].

### 3.2. Clinical Evidence

Clinical studies assessing the potential of PnDs in human tendinopathy have not been extensively conducted. However, the limited evidence that does exist leads us to believe that there is promising potential for this biologic in augmenting tendon healing.

Liu et al. conducted a controlled multicenter clinical trial with 89 patients who had flexor tendon injuries in zone II. They were divided into three groups (amnion, poly-DL-lactic acid (PDDLA), and control), with either hAM or PDDLA used to wrap the injured tendon repair in the experimental groups. The investigators found that the active ranges of motion of the interphalangeal joints, as well as the complication incidence in both experimental groups, did not significantly differ; however, they were significantly improved compared to the control group. Interestingly, they did find that the hAM group showed significantly better clinical outcomes when compared to the other groups [72].

### 3.3. Summary of PnDs in Tendinopathy

The current literature has uncovered the key factors that impact tendon healing, and there have been studies that have begun to identify the ways in which these factors can be manipulated by PnDs. Specifically, the inflammatory modulation of PnDs has most robustly been seen with hAM derivatives. Further, recent studies have uncovered the potential added benefit of utilizing a flowable PnD to access irregular spaces through a minimally invasive approach. Although we have made headway with in vitro and in vivo preclinical studies, there remains a need for further robust clinical research on the use of PnDs in human tendinopathy.

## 4. Nerve Injury

In addition to the traumatic etiologies of nerve injury, orthopedics exhibits the highest rates of iatrogenic peripheral nerve injury (PNI) due to surgical proximity to the nerves within the axial skeleton and the reconstruction techniques that are involved [67]. These pathologies are often devastating for patients, as pain, sensory loss, and even paralysis can result. After injury, Wallerian degeneration takes place and is followed by nerve regeneration [73]. This is an orchestrated process that includes the recruitment of macrophages and indigenous Schwann cells (SCs), phagocytosis of myelin, and the production of subsequent cytokines and chemokines [74,75]. Brain-derived neurotrophic factor (BDNF), glial-derived neurotrophic factor (GDNF), nerve growth factor (NGF), neurotrophins 3 and 4 (NT-3 and NT-4), and basic fibroblast growth factor (bFGF) are overexpressed by SCs lying at the distal segment to enhance neurite growth [73,74].

An effective nerve tissue engineering scaffold should possess biocompatibility, non-immunogenicity, and hemocompatibility [15]. It has been proposed that PnDs, specifically hAM, support the underlying neurological tissue through the production of neurotrophic factors like NGF [15]. Additionally, hAM scaffolds serve as a reservoir for many of these important neurotrophic factors involved in neuron survival [59,73]. This can allow for the PnD scaffold to be seeded with progenitor or stem cells to support nerve tissue growth and regeneration. The appropriate mechanical strength during healing, along with acceptable flexibility to prohibit the compression of the neural tissue, is also necessary. Ideally, this could be accomplished with a three-dimensional microstructure that is suitable for the optimal cell–surface interactions [73]. hAM is composed of a variety of collagens (types I-VII) and houses non-collagenous proteins such as elastin, laminin, and fibronectin [76]. These biomolecules are understood to provide strength and integrity to the amnion [59]. Their mechanical support has been utilized during instances of transferred tissue and nerve conduits that are involved in neurovascular reconstruction [59]. 

### 4.1. Preclinical Evidence

In vitro preclinical studies investigating PnDs and their impact on the biomolecular processes behind nerve healing have been conducted. Verdes et al. studied the impact of dhAM on nerve repair. dhAM supported stem cell adhesion, proliferation, and differentiation into the neural lineage. Specifically, the collagen, fibronectin, and laminin within the ECM promoted nerve cell adhesion and axonal elongation. Neurotrophic factors such as BDNF, NT-3, and NGF were also found to be elevated in dhAM-conditioned media, lending support to the claim previously made by Fairbairn et al. Additionally, these investigators found that differentiated neural-like cells that were seeded onto the dhAM adhered and grew on the basement membrane. Histological staining was confirmation, as it showed axons on the basement membrane that were integrated into the stroma of the dhAM [73].

Mligiliche et al. conducted an in vivo study using human amniotic matrix sheets as scaffolds for sciatic nerve regeneration in 24 rat models. These matrices were manufactured into various tubular constructs, called amnion matrix tubes (AMTs), which were then placed at the site of the created sciatic nerve injury and stitched repair. When assessing nerve regeneration and host reactions, the investigators did not identify much variation among the animals from each AMT group. Consistently, histologic examination revealed degradation of the tube wall by macrophages at week 3 and complete disappearance of the implanted AMTs by month 9. Additionally, electromyography was conducted and confirmed motor and sensory re-innervation at 9 months post-grafting. The EMG findings were comparable to those from the contralateral undamaged sciatic nerve [77].

Liang et al. studied axon regeneration in spinal cord injury (SCI) with the use of bone marrow stem cells (BMSCs) that were seeded onto denuded hAM. This was an in vivo study conducted using 54 adult rat models that were divided into five groups: 1. no SCI or treatment, 2. SCI alone, 3. SCI with denuded hAM, 4. SCI with BMSCs, and 5. SCI with denuded hAM + BMSCs. SCI was surgically induced with transection after a laminectomy at the T9 level. The groups receiving treatment with the denuded hAM had the matrix wrapped around the stumps of their spinal cords. In the hAM + BMSC group, the seeded surface faced the injury site. The BMSC-only group received a BMSC suspension via a glass pipette. The investigators measured the functional outcomes using the Basso–Beattie–Bresnahan (BBB) locomotor rating score and the cold spray test. In comparison to the other groups, the denuded hAM + BMSC group showed a significant BBB score improvement both 2 weeks and 10 weeks post-operatively. When assessing for superficial sensation with the cold spray test, the denuded hAM + BMSC group reflected the highest mean score, suggesting the best recovery compared to the other groups. Additionally, neuroanatomical tracing revealed that the denuded hAM + BMSC group had BDA-positive fibers both rostrally and caudally to the injury site, as well as GAP-43 immunoreactivity rostrally. Both BDA and GAP-43 were used as markers for axonal regeneration. FG labeling is also used as a marker for axonal regeneration, and five FG-labeled neurons were detected, in this group more than in any other group, in the sensorimotor cortex and brainstem. Lastly, immunohistochemistry identified expression patterns of neurofilament-H (NF-H), a regenerative marker for axons and neural-like cells, in both the BMSC-only and hAM + BMSC groups. This finding lends support to the ability of stem cell seeding onto denuded/decellularized matrices to promote the differentiation of BMSCs into neural cells [78].

Chen et al. built upon our understanding of the pro-regenerative and anti-inflammatory properties of hAM in peripheral nerve injury. These investigators added a gelatin nanofiber membrane (Gel) to dhAM and created a gel nanofiber–dhAM composite membrane to potentially counteract the rapid rate of degradation seen with dhAM alone. The sciatic nerve was surgically transected in rat models and subsequently repaired with sutures and wrapped with the membrane. At day 7 post-surgery, when compared with dhAM alone, the Gel–dhAM composite membrane had 35% less degradation. Further, biomechanical testing revealed a higher elastic modulus, strain to failure, maximum tensile strength, and suture retention strength in the composite membrane. Through an in vitro analysis, the Gel–dhAM membrane was found to reduce SC apoptosis and promote the proliferation of both the SCs and macrophages, both of which are vital components in the nerve repair process. When investigators assessed the regenerative outcomes of the nerve repair, the composite membrane was completely fused to the epineurium by week 4 and fully degraded by week 8. When examining the structure, the nerve had a uniform thickness and minimal adhesion formation, like that seen in an uninjured sciatic nerve. Further, the Gel–dhAM-treated nerves demonstrated more new continuous fibers for axon regeneration and resulted in restoration of the innervated muscle, like that of a normal muscle [79].

### 4.2. Clinical Evidence 

Clinical studies on the use of PnDs for the treatment of nerve injury have yielded positive outcomes and have begun to fortify the preclinical evidence that is already available on their effectiveness. Buentello-Volante et al. aimed to study the clinical outcomes of hAM transplantation in patients with median nerve injuries in carpal tunnel syndrome (CTS). The investigators conducted a single-arm randomized controlled trial where 35 patients with unilateral or bilateral CTS were enrolled. The patients underwent a carpal tunnel release surgery (CTRS), and those in the experimental group had a hAM fragment placed on the stromal side in contact with the median nerve. The control group did not receive a hAM fragment after CTRS. 

Functional outcomes were assessed through using the Boston Carpal Tunnel Syndrome Questionnaire (BCTQ). Although both groups showed a significant improvement at each follow-up post-operatively, the hAM group showed significantly better outcomes when compared to the control, starting at 6 months post-operatively and continuing to the conclusion of the study at 12 months post-operatively. Similarly, the symptom severity scale of the BCTQ showed the hAM group had significantly better outcomes at the same time points. The Disabilities of the Arm, Shoulder, and Hand (DASH) questionnaire was also administered to measure symptom severity. Its results followed the same trend and showed significantly better post-operative outcomes in the hAM group compared to the control at 6 months and 12 months. Lastly, the Historical Objective Scale, which measures the clinical severity of CTS, demonstrated that the hAM group had significantly lower severity scores at 6 months and 12 months post-operatively when compared to the control group [80].

Additionally, a case reported by Kalra et al. described a traumatic radial nerve injury due to an animal bite. A 3-year-old male suffered a monkey bite to his distal radial nerve 3 months prior and presented with wrist drop and an inability to extend the fingers and thumb of his right hand. Neurologic examination revealed complete paralysis of his brachioradialis muscle and wrist, digital, and thumb extensors, in addition to sensory loss of the dorsal first web space. High-frequency ultrasound and electrodiagnostic studies revealed a complete radial nerve injury. This patient was treated surgically with partial nerve end resection and epineural neurorrhaphy with a hAM nerve wrap at the site of repair.

Post-operatively, the patient was placed in a long-arm splint at 90 degrees for 6 weeks, followed by elbow mobilization exercises. The patient regained wrist extension at 2.5 months and finger and thumb extension at 4 months post-operatively. A three-level pain intensity scale revealed a pre-operative maximum score of 4, indicating “a lot of pain”. This improved to a score of 0, indicating “no pain”, at 6-month follow-up. The investigators concluded that the rate of nerve regeneration in this patient was faster than the conventional rate of 1 mm/day. This could be due to the healing potential of hAM, the lesser extent of perineural fibrosis, and/or the more robust regenerative ability of a young child [81].

### 4.3. Summary of PnDs in Nerve Injury

An ideal nerve repair material should inhibit inflammation, prevent adhesions, reduce scaring, and promote growth [82]. These characteristics have been seen in hAM PnDs, specifically in the form of membrane wraps. Varying forms of PnDs have been investigated, including a composite Gel–dhAM membrane which showed enhanced mechanical properties and a reduced degradation rate. Further, hAM was used as a scaffold onto which stem cells were seeded, promoting axonal regeneration. These novel directions show promise and offer clinical potential for the surgical treatment of PNI. However, positive clinical outcomes lack consistency in the studies currently found in the literature [83]. As a result, additional clinical investigation into these varying PnD applications is necessary.

## 5. Bone Healing

The rapidly growing rate of bone-related conditions and subsequent surgeries represents an urgent demand for new and effective bone graft materials [2,84]. These materials, autografts, allografts, xenografts, synthetic grafts, and growth factors, are used in many different applications, including the repair of fractures, spine surgery, and periodontal repairs [85,86,87]. The bone healing process, best understood in fracture healing, immediately follows injury and occurs through the phases of hematoma formation, soft callus formation, hard callus formation, and remodeling [88]. A hematoma forms at the site of the fracture due to a disruption in blood flow, and the cytokines and growth factors of the innate immune response and in the hematoma recruit cells such as mesenchymal stem cells and leukocytes to the area of injury [89]. Late in the inflammatory stage, macrophages are polarized into the M2 phenotype, and various growth factors that promote bone formation are released. These include TGF-β, VEGF, and bone morphogenetic proteins (BMPs), which recruit osteochondroprogenitor cells and stimulate their differentiation into osteoblast and chondrocyte lineages [90]. Then, the hematoma is replaced with a soft callus by chondrocyte secretion of cartilage. Osteoblast activity gradually results in mineralization, forming a hard callus. Newly formed bone matures as it enters the ongoing remodeling phase, characterized by cycles of resorption of bone by osteoclasts and deposition of bone by osteoblasts [88]. 

Based on the bone healing process, a good bone graft should (1) provide a matrix or substrate for cell attachment and mineralization; (2) promote cellular activity, such as the differentiation and proliferation of osteoprogenitors and osteoblasts; and (3) contain osteogenic and angiogenic growth factors. Important growth factors include the BMPs, TGF-β, and fibroblast growth factors (FGFs), which stimulate osteogenesis, and VEGF, which stimulates angiogenesis for the formation of new blood vessels in the healed bone. PnDs possess many of the qualities of a good bone graft. As discussed in previous sections, they can serve as scaffolds, which is especially important in bone healing for mineral deposition [91]. In particular, hACM contains a multitude of growth factors that are important for osteogenesis, including TGF-β1, bFGF, and PDGF [35]. Even acellular or decellularized PnDs contain growth factors that recruit cells and stimulate osteogenic activity in vivo, resulting in the presence of all three properties of an ideal bone graft material. Further, the anti-inflammatory properties of hAM and low immunogenicity of decellularized PnDs have also been shown to be beneficial in bone healing [92,93].

Research conducted using cells capable of osteogenic differentiation and activity suggests that bone graft materials containing certain PnDs may be osteoconductive, osteoinductive, and osteogenic. Animal defect models have been used to evaluate the regenerative capacity of these graft materials in vivo. A limited number of clinical studies have been conducted to determine whether these materials are safe to use and efficacious in promoting bone healing in humans. The following section focuses on select recent findings that show how PnDs may influence bone healing and its underlying processes. 

### 5.1. Preclinical Evidence

Go et al. performed a series of experiments that ultimately suggest that extracts of hCM are osteogenic in vitro. In their first study, they demonstrate that treatment of MG-63 cells (cultured in osteogenic induction medium) with hACM extract results in osteogenic differentiation (as compared to no treatment) based on the results of elevated alkaline phosphatase (ALP) activity; calcium deposition; the expression of markers such as runt-related transcription factor 2 (RUNX2), osteocalcin (OCN), and osteopontin (OPN); and mineralization [94]. Increased expression of ALP (a membrane protein) is indicative of early osteogenic differentiation. ALP plays a role in mineralization by increasing local concentrations of inorganic phosphate for mineralization [95]. OCN and OPN are expressed by mature osteoblasts and are secreted into the bone matrix; the former is especially specific to bone tissue [96,97]. Interestingly, this study found that hACM extract was superior to hAM extract in stimulating osteogenic activity. A follow-up study by the same group of investigators attributes this to differences in the growth factor concentrations between hAM and hCM extracts. Treatment of the same cell line with hCM extract resulted in significantly greater ALP activity, calcium content, expression of markers such as RUNX2 and OCN, and mineralization (as measured by alizarin red S staining). While the concentrations of bFGF and BMP-2 were found to be comparable in hAM and hCM extracts, the concentrations of TGF-β1 and epidermal growth factor (EGF) were significantly higher in the hAM extract (EGF was not detected in the hCM extract). Osteogenesis was found to be enhanced in cells treated with hAM and with an EGF inhibitor and inhibited in cells treated with hCM and EGF. hCM was additionally found to stimulate osteogenesis in human mesenchymal stem cells (hMSCs) [98]. The authors conclude that hCM can be used to promote osteogenesis due to its growth factor profile.

Other studies have utilized scaffolds incorporating perinatal derivatives that are seeded with cells and subsequently assessed for osteoconduction, osteoinduction, and osteogenesis. Kolliopoulos et al. utilized mineralized collagen scaffolds to investigate the effects of soluble and scaffold-incorporated hAM and hCM extracts on hMSCs. No significant differences in ALP activity were observed between treatment groups and a conventional mineralized collagen scaffold. Scaffolds incorporating either amnion or chorion released (prior to seeding) OPN, TIMP-2, and angiogenin. Soluble hAM was found to significantly increase the metabolic activity of seeded hMSCs over a period of 21 days. Scaffold-incorporated hAM increased the expression of OCN compared to the other treatments and conventional mineralized collagen scaffolds. Soluble and scaffold-incorporated hAM significantly increased BMP-2 expression. Scaffold-incorporated hAM and hCM significantly upregulated chemokine ligand 2 (CCL2), interleukin (IL)-2, and IL-8 compared to the conventional scaffold, soluble hAM extract, and soluble hCM extract. VEGF and angiopoietin 1 (both related to angiogenesis) were upregulated at various time points between day 3 and day 21 [99]. These results show that hAM and hCM alter the expression of key osteogenic and angiogenic genes in vitro. Further research is warranted to determine whether changes in chemokines such as CCL2 are associated with immunomodulation that positively affects bone healing.

A prior study conducted by Dewey et al. found that the incorporation of hAM into mineralized collagen scaffolds does not significantly enhance osteogenesis in vitro under normal conditions. Porcine adipose-derived stem cells (pASCs) seeded onto collagen-amnion scaffolds exhibited reduced metabolic activity and viability as compared to cells seeded onto conventional mineralized collagen scaffolds. The mineralization was comparable between groups at the final time point of the study. However, under an inflammatory challenge, cells seeded onto collagen-amnion scaffolds exhibited significantly increased expression of collagen and markers of osteogenic differentiation (BMP-2, RUNX2, and OCN) [100]. These results suggest that hAM (as part of a collagen scaffold) could potentially be used in bone healing applications where a chronic inflammatory response is predicted (for example, due to the material of an implant). 

Bone regeneration in vivo with bone grafts that incorporate hAM and hCM has been investigated using various defect models. Using a non-healing rat calvarial defect model, Dziedzic et al. assessed whether the application of four layers of dhAM with or without adipose-derived stromal cells (ASCs) supports bone healing as compared to no treatment. Cell differentiation was stimulated using an osteoblast differentiation medium. Decellularization of the hAM layers was confirmed by the lack of nuclei evidenced by H&E staining and did not affect the membrane structure. Microcomputed tomography (microCT) imaging after 12 weeks showed healing of the defect toward the central region in defects treated with dhAM (with and without ASCs). The dhAM with ASCs resulted in a significantly higher bone volume to total bone volume percent (BV/TV%) and trabecular number as compared to untreated defects. Histological imaging of the defects treated with dhAM showed clear evidence of osteoconduction and neovascularization [101]. A study by Fenelon et al. compared dhAM to fresh, cryopreserved, and lyophilized hAM treatments using a murine model with a diaphyseal bone defect. dhAM promoted early healing of the defect and was the only graft treatment that resulted in promoted bone formation one month following treatment [102].

PnDs may have the potential for use as auxiliary materials used to promote bone healing. Fenelon et al. found that incorporation of dhAM into a calcium phosphate cement (CPC) scaffold with BMP-2 did not significantly enhance the volume of bone formation in a rat femoral defect (as compared to CPC/BMP-2 alone) [103]. However, a more recent study evaluated the effect of hAM on heterotopic ossification using a critical-size diaphyseal defect model in rats. Priddy et al. found that surrounding a collagen sponge with hAM resulted in reduced heterotopic ossification and a reduced total bone volume as compared to a collagen sponge alone. At 12 weeks, the defect bone volume was not significantly different between groups. The authors suggest that molecules in hAM such as GAGs may retain BMP-2 and prevent its diffusion into soft tissue [104].

The method by which PnDs are delivered likely influences the effect they have on bone regeneration. Rameshbabu et al. developed a silk fibroin sponge that incorporated decellularized placental ECM (called PIMS). PIMS was compared as a bone graft material to plain silk fibroin (SF) scaffolds using a leporine model with a critical-sized tibial defect. PIMS was found to significantly increase BV/TV% at the defect as compared to SF (which significantly enhanced healing as compared to untreated defects). At the six-week time point, defects treated with PIMS showed comparatively mature mineralized bone tissue with osteons (not observed in the defects treated with SF). Additionally, using a chick chorioallantoic membrane assay, the investigators demonstrated significantly greater neovascularization with PIMS than with SF [105].

### 5.2. Clinical Evidence

Clinical studies on PnDs for bone regeneration have largely focused on periodontal applications. Venkatesan et al. conducted a randomized clinical trial (n = 50) comparing hAM and a collagen membrane as barrier membranes for guided tissue regeneration using a biphasic calcium phosphate graft. The probing pocket depth, clinical attachment loss, and bone fill were comparable between groups, suggesting that hAM can be used as a barrier membrane without negatively affecting the outcomes [106]. In another recent randomized clinical trial (*n* = 34), bony lesions in patients undergoing endodontic surgery receiving a hydroxyapatite bone graft were treated with either de-epithelialized hAM or platelet-rich fibrin over the bony crypt. Patients who received the hAM treatment developed significantly more vasculature than those who received fibrin; however, no differences in bone formation were observed [107].

Few clinical studies of the effects of PnDs on bone formation in non-periodontal applications have been reported in the literature. There is at least one currently enrolling study that is evaluating hAM for use in spinal fusion [108]. Brigido et al. report a case series of patients (*n* = 38 patients; 59 joints) who underwent hindfoot and ankle fusion in which an autograft mixed with allogenic placental connective tissue was used as the graft material. The most commonly treated joint in this study was the subtalar joint (24 joints); the time to fusion for this joint was 51.8 ± 17.9 days. The observed time to fusion, by joint and for all joints, was not compared to any reference values [109]. 

### 5.3. Summary of PnDs in Bone Injury

hAM and hCM appear to stimulate different aspects of osteogenesis and angiogenesis in vitro. The mixed results from preclinical studies may be due in part to the heterogeneity in PnD processing and the delivery method. However, further animal research on hCM as a bone graft material is warranted based on its osteogenic effects in vitro.

## 6. Osteoarthritis

Osteoarthritis (OA) is a progressive and degenerative joint disease characterized by degradation of the articular cartilage, remodeling of the subchondral bone, synovial inflammation, and osteophyte formation [110,111,112,113]. It is the leading cause of chronic disability and pain in the United States, costing $185 billion annually, and effects nearly 14 million Americans, with predictions of a 40% increase in its prevalence by 2035 [110,111,112,113]. However, research leads us to believe that the current treatment methods only manage the disease, with some evidence showing that corticosteroid injections can even cause a progression of degradation [110]. Degradation of the joint is driven by interactions between inflammatory mediators, primarily TNF-α and IL-1β [110,113]. The lack of control over these mediators leads to a catabolic effect, with a subsequent reduction in cartilage cellularity, a change in chondrocyte formation, and breakdown of the ECM of the joint cartilage [113].

As discussed previously, PnDs modulate inflammatory mediators and subsequently the inflammatory process as a whole. However, they have specifically impacted factors shown to cause the progression of OA, as well as resulting pain [112]. Recent evidence has shown that when hAM is applied to tissue, it exerts anti-inflammatory properties by downregulating the expression of CD80, CD86, and major histocompatibility complex class II antigens [34]. The following portions of this section will focus on the preclinical and clinical evidence on PnDs and their impact on the pathological processes of OA and subsequent patient outcomes. 

### 6.1. Preclinical Evidence

A decrease in the prominence of inflammatory cells gives rise to the possibility of cartilage preservation, if not regeneration. At the cellular level, Reece et al. provided strong evidence for the regeneration of cartilage with the addition of micronized dehydrated human amnion/chorion membrane (µ-dehydrated hACM). In this study, OA was induced in rats through medial meniscus transection (MMT) surgery, and different particle size distributions of µ-dehydrated hACM as µ-dehydrated hACM or reduced particle size µ-dehydrated hACM (RPS µ-dehydrated hACM) were tested. After being fluorescently tagged and tracked over 42 days post-surgery, protein elution from respective dilutions was quantified in vitro. There were 12 analytes that measured detectible levels, 4 of which were significantly different between the test groups: epithelial growth factor (EGF), fibroblast growth factor 2 (FGF2), platelet-derived growth factor BB (PDGF-BB), and chemokine ligand 5 (CCL5) [114]. The results showed that the standard size of µ-dehydrated hACM resulted in a decrease in protein expression. These data mirror previous literature showing that a decrease in inflammatory cells and inflammatory mediators can lead to less cartilage damage and, in some cases, restored cartilage [34,112,113]. In addition to the in vitro data provided by this study, the in vivo data showed a reduced lesion volume and a decreased cartilage surface roughness and osteophyte cartilage thickness and volume when compared to the control [114]. As for the particulate size comparison in this study, RPS µ-dehydrated hACM was outperformed by µ-dehydrated hACM in all metrics of its therapeutic effects [114].

In a similar study, Willett et al. performed an in vivo experiment using rat models to determine the effectiveness of µ-dehydrated hACM in staying within the synovial membrane and reducing cartilage degradation. After OA was induced in the study using MMT, data were collected at 3 and 21 days post-surgery. The study results indicated that µ-dehydrated hACM remained in the synovial membrane for up to at least 21 days post-injection, and erosion of the knee joint was significantly reduced. Their findings suggest that µ-dehydrated hACM injections can potentially have a therapeutic effect in OA [115]. 

Bhattacharjee et al. expanded upon these results when performing an in vivo study on male Sprague Dawley rats at 4-week time points. The four-pronged study involved inducing OA in rats via collagenase II injections. The OA-induced rats were injected with phosphate-buffered saline (control), adipose-derived stem cells (ADSCs), hAM gel, and ADSCs with the addition of hAM gel (hAM-ADSCs). They analyzed inflammation and cartilage regeneration by evaluating joint swelling, cytokine profiling in serum analysis, gross appearance, and histology. Arms that included the hAM gel showed a statistically significant decrease in knee diameter, a decrease in ICAM-1, leptin, and MCP-1, and an increase in TMP-1 at 28 days post-surgery. Additionally, the hAM gel arms also showed less visible cartilage damage when compared to the control and the ADSC-only groups. These results suggest that the addition of hAM to knees with OA could result in a significant decrease in inflammation and subsequent regeneration and maintenance of the cartilage [113]. 

**Figure 3:**
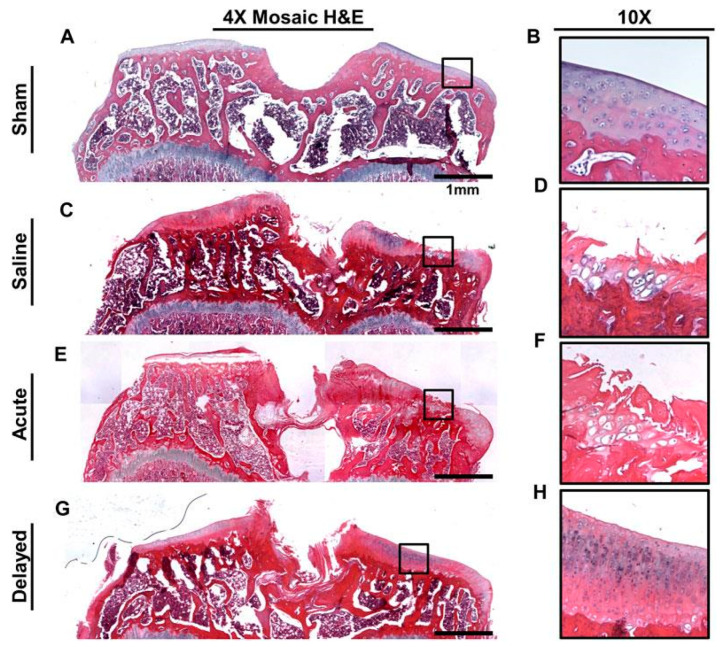
Histology results of OA-induced rats that received varying treatments. (**A**,**B**) Sham group displaying no damage; (**C**,**D**) saline group displaying cartilage damage on the medial plateau; (**E**,**F**) µ-dehydrated hACM group 24 h post-operative displaying cartilage damage; (**G**,**H**) µ-dehydrated hACM group 3 weeks post-operative displaying protection with no cartilage lesions. This figure is credited to [112] https://creativecommons.org/licenses/by/4.0/ accessed 30 May 2024; no alterations were made to the original figure.

Lin et al. continued upon the information provided by Bhattacharjee by elongating the study to a 6-week profile, while also delving into specific cartilage structural metrics. They also focused their study on the effectiveness of µ-dehydrated hACM. OA was produced through MMT, with sham surgery being performed to induce a control. OA-induced rats were injected with µ-dehydrated hACM either 24 h or 3 weeks after surgery. The study showed that knees treated with µ-dehydrated hACM were statistically significantly different when compared to the control in terms of their cartilage thickness, attenuation, surface roughness, and exposed bone area. Osteophyte degenerative changes were also shown to decrease with delayed injections of µ-dehydrated hACM. Delayed treatment groups showed statistically significant differences in comparison to the acute treatment groups in terms of bone mineral density and cartilage thickness when compared. These results were also consistent with the results from histology [112] (Figure 3).

### 6.2. Clinical Evidence

Due to substantial evidence of its effectiveness in vivo, several case studies have been performed to determine its efficacy in humans. In these studies, researchers presented outcome measures as patient-reported outcome measures (PROMs), the knee injury and osteoarthritis outcome score (KOOS), the Western Ontario and McMaster Universities Osteoarthritis Index (WOMAC)-A, or the reported analysis from blood or serum samples. The success rates vary; however, most studies show a significant improvement in the PROM scores. Vines et al. published a case series where six patients with symptomatic OA received a single intra-articular injection of micronized hAM and hAF [116]. Two patients had a transient pain increase; however, their overall PROM scores showed a general trend towards improvement [116]. Gellhorn and Han echoed these results by performing a larger case series of 40 patients with chronic tendinosis and arthropathy who received ultrasound-guided injections of dehydrated hACM into the intra-articular space [117]. The PROM scores were recorded at 1-, 2-, and 3-month time points, with a successful outcome being a 30% improvement in scores. At each time point, the patients reported a significant improvement in their pain and function PROM scores [117]. 

Castellanos and Tighe performed a single cohort study where human umbilical cord/amniotic membrane (hUC-AM) particulate was injected using ultrasound as guidance into the intra-articular space of 20 knees. The patients recorded their WOMAC-A scores at 6, 12, and 24 weeks post-injection. If the patient did not show a >30% reduction in pain at 6 weeks, they received a second AMUC injection. Pain was significantly decreased from the baseline to 6 weeks, 12 weeks, and 24 weeks, which coincided with a statistically significant improvement in physical function and stiffness at 12 weeks. It was determined that for the 11 patients who received a second shot at 6 weeks, this had a significant correlation with body mass index [111]. Alden et al. carried out a large retrospective case series where µ-dehydrated hACM was injected into the intra-articular space and followed up for 6 months. Patients reported their KOOS scores at 6 weeks and 3 and 6 months post-injection. Their mean KOOS score improved by 32, 56, and 65%, respectively, at each time point. Their pain scores improved by 67% at 6 months post-injection, while the quality and sports/recreation domains increased by 111% and 118%, respectively [118].

### 6.3. Summary of PnDs in Osteoarthritis

The PnD treatments discussed were shown to have anti-inflammatory effects, reduce pain, negate cartilage destruction, and promote cartilage regeneration. Specifically, hACM was the focus of a number of preclinical and clinical studies that yielded promising results in terms of these parameters, as well as in patient-reported outcomes. However, other studies found positive results utilizing other PnDs, such as hUC-AM particulate, hAM, and hAF. Although the preclinical data were corroborated with clinical evidence and lend validity to the potential use of hACM and other PnDs in OA, clinical data mainly came in the form of retrospective case series. Therefore, additional robust clinical research is needed to further confirm the findings discussed within the in vitro, in vivo, and clinical studies in order for substantiated recommendations to be made. 

## 7. Skeletal Muscle Injury

Skeletal muscles tissues are superficial and are frequently damaged, especially in sports, with an incidence ranging from 10% to 55% of all injuries [119]. A few common skeletal muscle injuries include contusions, strains, and, less commonly, lacerations [119,120]. The current approaches to the management of these different injuries are similar, with the use of the RICE principle (rest, ice, compression, and elevation), followed by a long course of physical rehabilitation. However, there is little scientific evidence to support these current practices.

Muscle tissue repair is a complex phenomenon involving degeneration, inflammation, regeneration, and fibrosis, which are continuous processes with significant overlap [121,122]. Several growth factors, as described by Baoge et al., can promote muscle regeneration, including bFGF, IGF, NGF, TGF-*β*1, and PDGF. Additionally, this regenerative process involves two key components. First, there needs to be myoblast proliferation and differentiation, driven by growth factors. Second, it is essential to minimize the amount of scar tissue formation [123]. TGF-β1 plays a pivotal role in muscle fibrosis formation during repair by stimulating cells to synthesize matrix proteins. After injury, TGF-β1 boosts the immune response, enhances cellular adhesion to the ECM, and prolongs myofibroblast survival by inhibiting apoptosis. Growth factors control satellite cell proliferation, differentiation, and fusion into myotubes both in vivo and in vitro. Recent findings indicate that they also promote myogenic cell differentiation, aiding in complete functional recovery post-muscle injury [123].

PnDs, specifically dhAMs, have potential as scaffolds for muscle repair, as they have been used as models to direct tissue reformation and create a suitable microenvironment [124]. Additionally, as previously discussed, PnDs can modulate the immune response and have trophic factors that are favorable for healing. They are also inexpensive and widely available. The decellularization of these products further reduces their immunogenicity and exposes endogenous proteins, making them an ideal candidate for tissue engineering [124].

### 7.1. Preclinical Evidence

Preclinically, there have been a few studies that have investigated the role of PnDs in treating muscle injury. Awad et al. sought to study the effectiveness of hAM and umbilical cord (AMUC) scaffolds in healing volumetric muscle loss in murine models. A quadriceps muscle defect was created in 15 mice divided equally into three groups: 1. an AMUC group, 2. an acellular collagen (ACS) group, and 3. an untreated control group. At the 8-week study endpoint, histologic analysis found that the AMUC group had a significantly higher cellular density than both the ACS and control groups. Upon immunofluorescence evaluation, the AMUC group had a significantly greater number of laminin and myosin heavy chains, indicative of greater organized muscle fiber formation [125].

Next, the investigators used magnetic resonance imaging and micro-computer tomography. After measuring some variance in the cross-sectional area and volume of muscle restoration, these differences were not found to be significantly different between the three groups. Further, gross clinical evaluation found that the AMUC group demonstrated the least amount of fibrosis (40% regenerated muscle) when compared to the ACS group (60%) and the control group (85%). Lastly, these gross findings were confirmed upon histological analysis, in addition to the AMUC group demonstrating a significantly higher cellular density as compared to the other groups [125].

Similarly, Izadi et al. conducted an in vivo study focused on the impact of a hAM scaffold with high-intensity interval training (HITT) on VML. Thirty-six rats had a surgical defect created in their tibialis anterior muscle and were randomly assigned into one of three groups: 1. dhAM alone, 2. dhAM + adipose-derived stem cells (ADSCs), and 3. untreated control. Then, each group was further divided into either a sedentary group or a HITT group to uncover a potential synergistic effect between treatment and rehabilitation. The expression levels of genes of neurotrophic factors were assessed due to their well-understood influences on skeletal muscle regeneration [126].

The researchers found that the dhAM + ADSC group who underwent HITT had significantly elevated brain-derived neurotrophic factor (BDNF) and neurotrophin-3 (NT3) mRNA levels when compared to the sedentary subgroup. Functional muscle testing was also utilized to assess the maximal isometric contractile force, which found that the maximal isometric force was also significantly increased within the dhAM + ADSC group when compared to both the dhAM-alone group and the untreated control group. Ultimately, the investigators found that the dhAM scaffold that was seeded with ADSCs, in conjunction with HITT, improved vascular perfusion, innervation, and muscle regeneration. This led to the conclusion that this biologic augmentation with HITT can enhance muscle regeneration after VML in rat models [126].

### 7.2. Summary of PnDs in Skeletal Muscle Injury

To our knowledge, no clinical studies have been conducted with PnDs to assess their efficacy in treating muscle pathology in humans. Current treatments are focused on the principles of RICE, along with the use of non-steroidal anti-inflammatory drugs, which carry their own level scientific controversy. Although novel therapeutic approaches aimed at modulating growth factors, such as PnDs, can potentially enhance muscle repair and have significant clinical applications, this conclusion cannot be made with the current available evidence. Further studies are needed for more robust scientific investigation within this space.

## 8. Adhesions

Pathologic adhesions occur in up to 95% of all surgeries but are mostly associated with tendon, pericardial, abdominal, epidural, and intrauterine procedures [127,128]. Due to their relevance in orthopedic surgery, we will review the underlying biological processes and potential benefit of PnDs in their treatment. The focus of this section will be the most common adhesions in surgical applications, including tendon and nerve adhesions. These are one of the most common failure modalities in procedures such as peripheral nerve repair, carpel tunnel release, and lumbar laminectomy [80,129]. The formation of fibrous and scar tissue, which leads to pathological adhesions, is the result of a combination of biological factors, including inhibition of the fibrinolytic system, disruption of ECM degradation, a sustained inflammatory response, and tissue hypoxia, which leads to increased angiogenesis [128]. 

Several cell types and cytokines have been identified as key contributors to this process. For example, fibroblasts are responsible for collagenous fiber and granulation tissue formation, and their proliferation is dependent in part on the presence of TGF-β1, FGF, and IL-6 [128]. Neutrophils and macrophages may contribute to the formation of pathological adhesions when in their “activated” state. The concentration of other cytokines, such as IL-1β, TNF-α, and VEGFa, has also been directly correlated with the degree of adhesion formation [128].

The current strategies for preventing pathological adhesions include fat transplants or transplants of other synthetic products to serve as a physical barrier. Ideally, this barrier will prevent the ingrowth of fibrous tissue while allowing for nutrient transport and nerve or ligament taxis [32]. Perinatal derivatives may also represent a viable solution. Allograft products derived from perinatal tissues are known to contain proteins, growth factors, and cytokines which inhibit scar formation and have immunomodulatory and anti-inflammatory effects [73,130]. Certain perinatal derivatives such as hAM are also semipermeable, allowing for nutrient diffusion to the tissue of interest while preventing fibrous tissue ingrowth [70]. In addition to providing a protective measure, perinatal derivatives have been shown to aid in tissue regeneration as well given that decellularized perinatal derivatives such as hAM, hACM, and hCM are rich in growth factors associated with tendon and nerve repair [73].

### 8.1. Preclinical Evidence

To maximize the performance of PnDs, it is important to understand how the product processing and packaging could impact cellular interactions with the material. The amniotic membrane has an epithelial layer and a stromal layer, separated by a basement membrane [15]. Experiments conducted with human fibroblasts showed significant differences in cellular attachment and proliferation based on when the cells were exposed to the epithelial or stromal layer of fresh amniotic membrane samples, with the epithelial side allowing for significantly less fibroblast adhesion. When extrapolated to a rat sciatic nerve scarring model, this translated into significant reductions in perineural adhesions, fibrosis, and scarring 4 weeks after neurolysis procedures. These reductions had a meaningful effect on functional performance as well, with the animals in the hAM group demonstrating significant improvements in nerve function compared to control at the same time point [32]. Similar studies have demonstrated that decellularized and dehydrated tissues also produce meaningful improvements in the prevention of surgical adhesions.

Processing of PnDs has a significant impact on the degradation characteristics of the material. Techniques such as crosslinking with glutaraldehyde or 1-ethyl-3 (3-dimethylaminopropyl) carbodiimide (EDC) can improve the graft survival time and increase its biomechanical strength [15]. Improving the stability of graft products can have a significant impact on the final outcomes, as illustrated in the 2009 study from Tao and Fan, which compared freeze-dried and cross-linked amniotic membranes to autologous free fat transplantation and a negative control. While the cross-linked cohort demonstrated significant improvements in scar formation and adhesion tenacity in a canine model of post-laminectomy epidural fibrosis, the freeze-dried cohort failed to demonstrate any improvement relative to the negative control. Histological evaluation indicated that while both products provided protection to the spinal cord at 1 week post-laminectomy, by the 6-week time point, the freeze-dried amnion samples had undergone significant degradation, with only small fragments remaining [131]. 

Cunningham et al. reported on a similar post-laminectomy epidural adhesion study in an ovine model, demonstrating that a dual-layer, chorion-free amnion patch was able to produce significant improvements with respect to adhesion tenacity and fibroblast activity [132]. The product used in this study was likely produced through the lamination of two amniotic membrane sheets such that the stromal sides of the amnion were together, followed by subsequent dehydration [15]. While the authors did not comment on the degradation rate of the amniotic graft used in this study, it appeared to be sufficient in preventing adhesion formation. This study also supports the work by Lemke at al. indicating that the epithelial side of the amniotic membrane is effective in reducing fibroblast activity. Cunningham et al. did not find a significant difference in the fibroblast number between the DLAM cohort and the negative control; however, they did report a significant decrease in fibroblast infiltration into the dura mater. This indicates that the amniotic membrane is effective in preventing adhesion formation, particularly when the epithelial layer is positioned correctly [32,132]. 

The immunomodulatory potential of PnDs is hypothesized to be one of the major factors leading to these improvements in outcomes. Another rat model of sciatic nerve injury established in 2020 by Dong et al. was used to evaluate the potential of PnD products in reducing adhesions and promoting nerve repair. This study also showed significant reductions in nerve adhesions by 4 weeks post-operation and showed these benefits were maintained out to 12 weeks. The authors reported a significant reduction in M1 macrophage expression and elevated nerve growth factor levels [82]. Other preclinical nerve studies have shown that in addition to reducing macrophage activation (M1 expression), hAM grafts can also decrease the expression of inflammatory cytokines such as IL-6 and TNF-α, while also increasing the expression of M2 macrophages and anti-inflammatory cytokines IL-10 and IL-13, when used to protect neural structures [133]. 

### 8.2. Clinical Evidence

A 2022 clinical trial by Wang et al. observed significantly lower inflammation and subsequent adhesion formation when using amnion grafts compared to a negative control in the treatment of intrauterine adhesions. After hysteroscopic adhesiolysis procedures, freeze-dried amnion grafts were placed epithelial side outward within the uterine cavity. The post-operative re-adhesion rates and the concentrations of TNF-α, IL-1β, and VEGF (vascular endothelial growth factor) from intrauterine exudate samples were quantified at multiple time points. The authors saw a significant reduction in all three cytokine levels at all eleven time points from 3 h to 7 days post-operation when comparing the amniotic membrane cohort to the control (n = 15 each). These reductions in inflammation translated into a significant decrease in re-adhesion rates during reoperations at 3 months after the initial procedures [129]. While inflammatory cytokines such as TNF-α, IL-1β, and VEGF are a necessary part of the healing process, overexpression of these factors can lead to imbalance in the formation and breakdown of the extracellular matrix. The available data in the literature, both preclinical and clinical, indicate amniotic membrane grafts can reduce this inflammation without interfering with the normal healing process. 

Controlling inflammation may also lead to measurable differences in patient pain levels and function. A 2020 clinical trial illustrates these improvements relative to carpal tunnel release procedures. Patients with either bilateral or unilateral carpal tunnel syndrome (CTS) for whom conservative care had failed had carpal tunnel release surgery with or without the use of an amniotic membrane graft. Frozen membranes were placed such that the stromal side of the membrane was in contact with the median nerve. The authors found significant improvements in functional status, symptom severity, Disabilities of the Arm, Shoulder, and Hand (DASH) scores, and historical objective scale scores for both cohorts at 6- and 12-month follow-up intervals. However, there was significantly more improvement in the amniotic membrane cohort relative to the control across all metrics, leading the authors to conclude that CTRS with the inclusion of amniotic membrane transplantation is superior to CTRS alone [80]. 

Similarly, Gaspar et al. illustrated these improvements by investigating the use of a commercially available dehydrated amnion graft in the treatment of recurrent cubital tunnel syndrome. The ulnar nerve of eight patients with at least two prior surgeries was wrapped circumferentially with the graft and rehydrated with saline. Patients were then followed up for an average of 30 months post-operatively, with all patients showing significant improvements in their VAS (Visual Analogue Scale) pain, grip strength, and QuickDASH outcomes [134]. A larger trial was conducted by Clayman et al., who examined the potential of amniotic membrane grafts in preventing laryngeal nerve injury during thyroid surgery. A total of 100 consecutive nerves from 67 patients were wrapped with a commercially available amniotic membrane graft immediately after dissection to prevent desiccation and contact with surgical instruments. This patient sample was compared to a matched control of 134 patients with respect to signs of RLN injury such as dysphonia, aphonia, difficulty breathing, and difficulty swallowing. At 24 h after surgery, there were signs of transient nerve damage in 12.5% of the control cohort compared to only 1.5% in the amniotic membrane cohort. Furthermore, there were no reports of nerve injury in the amniotic membrane cohort at 21 days, while the control cohort reported an injury rate of 5%. While pain was not objectively assessed in this study, the authors did note that fewer reports of pain were observed in the amniotic membrane cohort [130].

### 8.3. Summary of PnDs in Adhesions

PnD graft products have the potential to significantly reduce the burden of pathological adhesions in a wide range of surgical procedures. Preclinical studies have shown how these grafts could reduce the effectiveness of fibroblasts and result in decreased adhesions. Similarly, in clinical studies, operative application of these biologics may be beneficial in preventing adhesion formation. Graft survivability is also an important consideration, with some evidence that frozen tissues may degrade too quickly to fully prevent adhesion formation. Further research may be warranted to determine which types of commercially available graft products have the appropriate degradation characteristics for all applications.

## 9. Discussion

Based on their ability to promote the regeneration of a wide range of tissues, PnDs have the potential to be applied to challenging musculoskeletal conditions—especially those for which there are currently no highly effective treatments. For example, VML injury is generally treated with physical therapy, which has shown limited efficacy in promoting functional recovery [135]. Similarly, skeletal muscle hamstring injuries are often treated conservatively with RICE. Alternative options such as platelet-rich plasma injections have shown mixed results in the literature [136]. More common MSK pathologies may benefit from PnDs as well, as these conditions still exhibit a considerable burden of disease, despite advances in treatment (e.g., wounds, tendinopathy, nerve injury). 

Although some pathology-specific variation exists, the progression of most of these conditions follows a similar pattern of injury, inflammation, proliferation, and remodeling [73]. The underlying mechanisms are driven by an orchestrated inflammatory response and rebuilding process that is carried out by the effects of critical cytokines and growth factors. Further, the tissue-specific cells (e.g., tenocytes, neurites, chondrocytes) may be unique to each pathology; however, certain biomolecules have been found to be a common thread. For instance, the TGF-B superfamily is well understood to be significantly involved in developmental processes [137,138]. A few of their roles include regulating cellular differentiation and proliferation, while also modulating osteogenic, chondrogenic, and tenogenic development [137,138]. Similarly, FGFs play an important role in regulating tissue homeostasis, while PDGF is a powerful activator of the mesenchymal cells [139,140]. PnDs house many of these critical growth factors and, by interacting with the tissue-specific environment, have displayed the potential to augment and accelerate tissue healing. First, their anti-inflammatory effects are readily apparent in preclinical studies which have shown the downregulation of TNF-a in wound and OA models, decreases in MMPs in tendinopathy, and increased expression of M2 macrophages in adhesion models [42,57,101,103,123]. There is also strong evidence of their growth-promoting effects, as nerve injury studies with PnDs have shown the elevation of neurotrophic factors (e.g., NGF, NT-3, and BNDF) and bone osteogenesis investigations have found significantly increased BMP-2 expression in their hAM-treated cohorts, to name a few [63,89]. 

However, the composition and concentration of growth factors vary by the type of PnD (e.g., hAM, hCM, or hACM). Table 2 provides a brief summary of the composition of the most commonly discussed perinatal components and derivatives in this review. In addition to the variation due to PnD type, there is variation due to the way in which these biologics are processed; processing should be a consideration for physicians when determining the optimal treatment. Once isolated from the placental tissue, PnDs can either remain fresh or become decellularized [15]. The decellularization process has garnered attention due to yielding products with low immunogenicity by removing native cells and leaving an acellular scaffold of the ECM that retains proteins and growth factors [15]. These biologics also undergo varying preservation methods, including cryopreservation, lyophilization (e.g., freeze-drying), and dehydration, each with its own benefits and drawbacks. For instance, cryopreservation may preserve growth factors and proteins better when compared to lyophilization, but the latter may maintain antimicrobial activity and structure better [14,26]. Dehydration, when compared in studies to the cryopreservation of PnDs, has resulted in a disrupted ECM [14,71]. As with any allograft product, sterilization is an important part of the processing due to the risk of communicable disease. The two most used methods are gamma irradiation and peracetic acid, specifically in the context of hAM. The impact of these processes on the availability and bioactivity of various endogenous growth factors in these products is not well understood, and more research is needed to fully understand their impact [15].

When considering PnDs for orthopedic applications, it is imperative to take not only the specific PnD into consideration but also its subsequent processing. For surgical and traumatic wound healing, the antibacterial properties that hAM provides, in addition to its anti-inflammatory and scaffold characteristics, makes it a potentially favorable choice. While hAM has also been positively validated as a subcutaneous graft, its incorporation into orthopedic surgery is more applicable as a surface dressing for surgical incisions. Further, both fresh and decellularized PnDs have displayed promising results as processed hydrogels and as intact biologics. Although there has been a committed effort to investigate the viability of PnDs within wound healing, orthopedic applications still require further investigation before clinical recommendations can be made. In tendinopathy, in vitro and in vivo studies have shown positive results using a variety of PnDs (hAM, hACM, and dhUC-WJ) as tendon wraps at the site of injury. However, these PnDs underwent vastly different processing, and there remains a gap in our understanding of how these differences translate into tendinopathy clinically [68,69,70,71,72]. Next, within the nerve injury space, ample research has shown hAM promotes axonal regeneration and drives stem cell differentiation when acting as a scaffold [69,70]. The clinical findings have suggested accelerated healing properties and improved clinical functional outcome scores when it is used as a wrap [71,72]. Of note, hAM was given FDA approval to be used as a nerve wrap or conduit for PNI, adding an additional layer of support for its use [141]. The range of processing methods, which has led to studies with gel nanofiber composite membranes, amniotic matrix tubes, and decellularized scaffolds, provides promising insight into these varying applications. However, like tendinopathy, this necessitates caution, as further clarity on and consistency within PnD processing are needed for physicians to make the most informed treatment decisions.

As bone-related conditions and complications are on the rise, so is the effort to find effective PnD biologics [2,74,132,133]. hAM has been demonstrated to be an effective barrier membrane for guided tissue regeneration in periodontal applications. The findings of studies conducted in animal models and humans suggest that hAM does not impair bone formation and may have the potential to be applied in an auxiliary role to promote bone healing. For example, hAM may be beneficial in bone grafting situations where there is a high risk of chronic inflammation or where soluble growth factors are utilized that need to be retained at the healing bone tissue. However, identifying their ideal clinical use as an extract, a cement scaffold, or a novel silk fibroin sponge requires more investigation. For OA, the literature offers support for a wide range of PnDs, including hACM, hAM, hUC-AM, and hAF, due to their anti-inflammatory properties. The preclinical evidence has provided insight into their cartilage regeneration potential and ability to reduce osteophytes [103,104,105]. Further, clinical studies with intra-articular injections have shown improvements in patient-reported outcomes, functional outcomes, and pain scores up to 6 months post-injection [80,101,107]. Micronized dehydrated injectable forms of these PnDs are well represented and supported within the literature. However, definitively identifying the optimal PnD in terms of the processing and tissue source requires further investigation.

In skeletal muscle injury, preclinical studies have begun to investigate hAM as a bio-scaffold in animal models. Although they are limited, investigations have focused on VML and the impact of supplemental HITT in combination with PnD treatment. Early findings suggest the use of this PnD could result in more organized muscle fiber formation and ultimately muscle regeneration [115,116]. However, more robust preclinical and clinical investigations are necessary to draw conclusions about using PnDs in this space. Lastly, the most used PnD for pathologic adhesions is hAM as a biologic graft. Both commercially available grafts which have been minimally manipulated and investigational products using fresh tissues have demonstrated efficacy in reducing adhesion formation and inflammation and improving function in preclinical studies [32]. Certain clinical studies have also found patient functional scores and pain scores to improve with the use of this PnD graft in surgery [80,105,134]. Clinical study findings have also suggested that the directionality of the graft may be an important consideration, with the literature favoring placement of the stromal side of the membrane in contact with the tissue. Potential challenges in upscaling the production of PnD for clinical use include ensuring consistent directionality of the membranes, developing quality control methodology to ensure consistency of the product composition, and tailoring the production of PnDs to their most suitable applications. Ultimately, as is consistent with other MSK pathologies and sequelae, there remains a gap in the literature in identifying which processing methods are most effective for the respective tissue environments. 

The potential use cases of PnDs, stem cell therapies, and growth factors (such as those produced as recombinant proteins) significantly overlap. Stem cell therapies utilize human pluripotent stem cells, multipotent stem cells, or progenitor cells. These cells are delivered to the treatment site by injection or by implantation of a scaffold upon which the cells have been seeded [142]. The most common stem cell application in recent history has been hematopoietic stem cell transplantation for certain cancers or autoimmune diseases. Implanted cells facilitate the production of healthy blood cells in the host [143]. Recombinant growth factors, on the other hand, can be used for targeted action due to their purity and specificity. For example, rhBMP-2 is used in bone healing applications (such as spine fusion) due to its osteogenic potential [144]. While a variety of products in each of these categories (PnDs, stem cell therapies, and growth factors) are currently available on the market, PnDs offer unique advantages that favor their application in tissue regeneration applications, including those in orthopedics. Table 3 presents the advantages and disadvantages of each of these products in tissue regeneration applications. 

Although processed PnDs may not contain viable cells, they serve as a scaffold for endogenous cell growth and a rich source of growth factors that can facilitate the recruitment, differentiation, and proliferation of cells. Non-viable PnDs are thus capable of bringing about cellular activity conducive to tissue regeneration. Furthermore, the growth factors present in PnDs are likely present within, or close to within, physiologic concentration ranges. Certain growth factors, like rhBMP-2, must be used at high (or supraphysiologic) doses for efficacy, which has been shown to result in side effects such as heterotopic ossification [145]. The diverse set of growth factors present in PnD (which promote the development of the fetus in utero) may better orchestrate tissue regeneration than the administration of a high dose of a single growth factor. Ultimately, randomized controlled trials comparing PnDs to these alternatives will be needed to determine the appropriate indications for treatment with these products.

### Future Directions

The newfound interest in PnD biologics has sparked an exponential growth in the research. With these increased efforts, there has been inconsistency in the way in which PnDs and their production are described in the literature. Our review followed the guidelines reported by Silini et al., who were supported by COST, for standardizing the nomenclature of the various PnDs investigated. However, we hope that there will be more clarity behind the processing of PnD biologics to better differentiate each product. Additionally, we encourage the commercially available PnDs to improve their labeling and product descriptions, as there is a lack of specifying information that would further identify the PnD composition. This is problematic because of the diverse options available in this space and the pathology-specific benefits that the different PnDs hold. Several PnD biologics are already available on the market. However, there remains a need to standardize the processing methods and increase product transparency to promote broad acceptance and use of these biologics. We hope that these efforts are undertaken by both researchers and biologic companies to promote clarity so that physicians are best able to recommend specific PnD treatments to their patients. 

## 10. Conclusions

In this review, we discussed how PnDs offer properties that can augment the healing process in a range of common orthopedic MSK pathologies. Among the different applications, immune modulation was vital for healing in each. PnDs were found to influence the natural balance between anti- and pro-inflammatory factors and, in other contexts, acted as biologic scaffolds for further growth. The varied options in terms of the type and form of the PnDs were also explored by identifying the gaps that remain in our understanding, especially in terms of the biologic processing methods. Although some orthopedic conditions have more support for use of PnD biologics in the literature than others, more robust clinical studies, in the form of trials, are needed to fully investigate the clinical impact of these biologics in humans. 

## Figures and Tables

**Table 1:** Amniotic membrane angiogenic growth factors and mechanisms.

Growth Factor	Impact on Angiogenesis	Source
Angiogenin	Exerts ribonucleolytic activity, binds to membrane actin to induce basement membrane degradation, binds to a putative 170 kDa protein and transduces the signal into the cytoplasm, and directly enhances ribosomal RNA transcription in target cell nuclei	Gao, 2008 [37]
Angiopoietin-2	Activates the Tie2 pathway	Thurston, 2012 [38]
Epidermal growth factor	Upregulates VEGF expression	Cruijsen, 2005 [39]
Basic fibroblast growth factor	Induces an angiogenic fibrocyte phenotype in granulation tissues	Nakamichi, 2016 [40]
Heparin-binding epidermal growth factor	Activates the PI3K, MAPK, and eNOS pathways	Mehta, 2007 [41]
Hepatocyte growth factor	Upregulates VEGF expression and amplifies VEGF-driven angiogenesis	Xin, 2001 [42]
Platelet-derived growth factor	Upregulates VEGF expression and modulates the proliferation/recruitment of perivascular cells	Raica, 2010 [43]
Placental growth factor	Affects endothelial cells directly through VEGFR-1, creates VEGF from VEGFR-1, recruits monocytes/macrophages, and mobilizes hematopoietic progenitor cells from bone marrow	Ribatti, 2008 [44]
Vascular endothelial growth factor	Enhances endothelial cell proliferation, survival, migration, and permeability	Niu, 2010 [45]

**Table 2:** Comparison of select perinatal components and derivatives.

Component/Derivative	Tissue Type	Select Growth Factors and Cytokines Discussed in This Review	Example Use Case(s) in Orthopedics	References
**hAM**	Amnion membrane	Angiogenin, angiopoietin-2, epidermal growth factor, bFGF, PDGF, VEGF, EGF, OPN	Wound healing	[27,33,34,37,38,39,40,43,45,68,98,99]
**hCM**	Chorion membrane	OPN, TIMP-2	Guided tissue regeneration for periodontal reconstruction	[99]
**dhAM**	Amnion membrane (dehydrated)	IL-8	Muscle repair, peripheral nerve injury	[35,99]
**dhACM**	Amnion chorion membrane (dehydrated)	IL-4, IL-10, IL-6, PDGF, b-FGF, ILGF-1, TGF-B1, OPN, TIMP-2	Wound healing	[28,35,36,68]

**Table 3:** Comparison of potential advantages and disadvantages of PnDs, stem cells, and growth factors in tissue regeneration applications.

Product	Example Applications	Potential Advantages	Potential Disadvantages
**PnDs**	Soft tissue barrier Wound coverage Nerve wrapping Tissue grafts for wound care	Immunomodulation Provide both a scaffold for cell growth and a large range of growth factors Safety (as perinatal tissues are immune-privileged) Availability Cost	Limited potency Variability in quality Variability in growth factor composition Regulatory issues Social acceptance
**Stem Cells**	Hematopoietic stem cell transplantation to treat certain cancers and autoimmune diseases Repair of damaged heart muscle tissue Treatment of amyotrophic lateral sclerosis using autologous mesenchymal cells Treatment of spinal cord injuries using mesenchymal cells Intradiscal injection of stem cells to treat degenerative disc disease	Regenerative potential Versatility due to pluripotency Larger body of research Exact study design/external and internal validity	Adverse effects Cost Ethical issues and social acceptance Regulatory issues
**Growth Factors**	Osteoinductive application of rhBMP-2 Granulocyte colony-stimulating factors Recombinant nerve growth factor for eye disease (current area of research)	Purity and potency Feasibility of quality control Low product variability Specificity Used in combination with other therapies (such as bone grafts)	Stability Short half-life in vivo (potential need for repeated treatment) Cost Side effects due to dosing

## Data Availability

Not applicable.
